# P-2263. Are you PRePared After Transplant?

**DOI:** 10.1093/ofid/ofae631.2416

**Published:** 2025-01-29

**Authors:** Jylian Grabell, Aaron D Mishkin, Jacqueline Burnell, Eric Altneu

**Affiliations:** Lewis Katz School of Medicine Temple University, Philadelphia, Pennsylvania; Lewis Katz School of Medicine at Temple University, Philadelphia, Pennsylvania; Temple University, Philadelphia, Pennsylvania; Temple University Health System, Philadelphia, PA

## Abstract

**Background:**

Solid organ transplantation (SOT) is a life-saving medical procedure that can improve the quality of life of individuals with end-stage organ failure. Given the scarcity of organs, it is important to safeguard those implanted and prevent infections. Transplant Infectious Disease physicians help mitigate infectious risk both before and after surgery. One important preventable infection is Human Immunodeficiency Virus (HIV). Pre-exposure prophylaxis (PrEP) is effective in reducing the risk of HIV infection. Yet no study has examined PrEP use in SOT patients. The CDC reports that 30% of patients that may benefit from PrEP are prescribed them in the US. No similar statistic exists for SOT patients.

Figure 1

**Figure:**
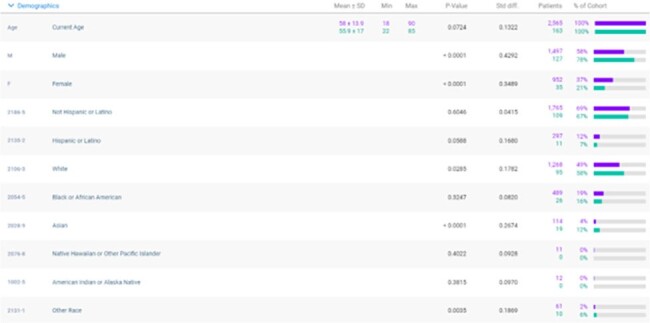

Demographics for SOT patients with possible exposure to HIV. Purple represents those without PrEP. Green represents those with PrEP.

**Methods:**

To obtain quantitative support for further study, a large, multi-center database (Trinetx) was queried. Trinetx contains deidentified data from hundreds of international healthcare organizations (HCOs) dating back about seven years. The retrospective query included 68 HCOs with patients that met the following search inclusion criteria (based on ICD codes): SOT patient without the diagnosis of HIV, prescribed PrEP, and possible exposure risk for HIV.

**Results:**

147,145 SOT patients were identified that did not carry the diagnosis of HIV. Only 160 patients (0.11%) were prescribed PrEP, identified as the following three regimens: tenofovir alafenamide + emtricitabine, tenofovir disoproxil + emtricitabine, or cabotegravir.

To risk-stratify HIV exposure, the original cohort of 147,475 SOT patients without HIV were analyzed with ICD codes for HIV exposure. This search identified 2,565 patients coded for HIV exposure. Given that only 160 patients were prescribed PrEP in the cohort, 2,405 patients may have benefited from PrEP but were not prescribed these medications. Patients on PrEP after SOT were predominantly male (78%) and White/Caucasian (58%). Identifying as Asian was a significant factor in using PrEP after SOT (p< 0.0001). Female gender was a significant demographic not utilizing PrEP despite possible exposure to HIV (p< 0.0001). (Figure 1).

**Conclusion:**

This large database study shows that PrEP is underutilized in SOT patients. Further study—including surveys of sexual practices, provider knowledge, and attitudes towards PrEP, as well as single-center analysis—is needed.

**Disclosures:**

All Authors: No reported disclosures

